# Supplementary material for: Are carbon emissions trading and green financial instruments synergistic? -Comprehensive quantitative research based on content analysis
Source: PLoS One. 2024 Mar 7;19(3):e0298601. doi: 10.1371/journal.pone.0298601 (PMC10919601; doi:10.1371/journal.pone.0298601)
Supplement: S1 Data — (ZIP) [file pone.0298601.s001.zip › Data/地级市Z_F工作报告.zip!/─┌├╔╣┼/╬┌║ú╩╨/└┤╫╘╣1⁄2╓┌║┼í╛┬φ┐╦╩2╛▌═°í┐_2014.docx]

各位代表：

　　现在，我代表市人民政府向大会报告工作，请予审议，并请市政协委员和列席会议的同志们提出意见。

　　一、2013年工作回顾

　　2013年，在自治区党委、政府和市委的正确领导下，在市人大、政协的监督支持下，全市上下认真贯彻落实党的十八大精神和自治区“8337”发展思路，深入实施“一个中心、两个转型、三个率先、五个乌海”发展战略，凝心聚力、开拓创新，经济社会发展保持稳中有进的良好态势。

　　（一）经济总体保持平稳运行。预计全年地区生产总值完成600亿元，增长10.5%;固定资产投资完成416亿元，增长20%;社会消费品零售总额完成113亿元，增长12.5%。地方财政总收入完成102.7亿元，增长8.6%;公共财政预算收入完成 68.5亿元，增长26%。园区经济不断壮大。预计园区工业总产值达到760亿元，增长12%，居全区27个重点工业园区第3位。出台了加快工业转型升级和工业园区建设的意见，进一步明确园区发展定位和产业转型升级方向。全年投入9.5亿元，进一步加大产业转型升级配套设施建设力度，园区承载能力显著提升。全面启动乌海国家级经济开发区和环乌海湖高新技术产业开发区申报工作，乌达工业园区被评为自治区新型工业化示范园区，海南工业园区被评为自治区精细化工产业示范基地。非公经济快速发展。出台了鼓励和支持非公经济发展的意见，进一步加大对非公企业的扶持力度。预计非公经济增加值完成396亿元，占GDP比重66%，非公企业解决了74%以上新增就业人口，非公经济成为拉动经济增长和促进就业的主要动力。招商引资成效明显。全年实施招商引资项目202个，引进到位资金271亿元，增长13%，争取上级各类资金30亿元。

　　（二）产业结构调整不断深化。工业经济转型步伐加快。预计工业增加值完成380亿元，增长12.5%;规模以上工业增加值增长13%;工业固定资产投资完成245亿元，增长22.5%。主导产业规模不断壮大。煤焦化、氯碱化工两大基地优势进一步显现，焦炭产能达到1800万吨，PVC产能达到120万吨;焦炭和PVC产能占全区比重分别达到60%和40%;全年利用市外煤炭资源1600万吨，占煤炭利用总量的40%。清华紫荆贝氏体钢项目正式签约，包钢万腾节能环保技改二期进展顺利，具备120万吨生铁、120万吨钢和120万吨轧材生产能力。维斯通管业PE管材、蓝星低辐射镀膜玻璃搬迁改造项目建成投产。全市电力装机容量达到360万千瓦，增长5.9%。全年完成电力多边交易45亿度，为企业补贴电费8000万元;减免煤管费、安排技术进步扶持等资金3.65亿元，支持企业正常生产和技术创新。产业延伸升级成效显著。煤焦油深加工能力达到90万吨，管材型材、塑料薄膜等PVC深加工能力达到12万吨。神华硝铵、华油焦炉煤气制天然气二期、君正煤矸石热电联产项目基本建成，宝化万辰煤焦油深加工、黑猫三兴精细化工等项目加快建设，煤焦化工逐步实现由以焦为主向以化产为主转变。东源科技一期1,4-丁二醇、腾龙生物农药中间体等项目建成试产，宜化季戊四醇项目主体建成，乙炔化工产品逐步实现由以PVC为主向聚乙烯醇、1,4-丁二醇等扩展延伸。产业多元发展步伐加快。格瑞特选煤设备、冀东改装车等装备制造项目建成，海能超细纤维、天宇高档填料二期等新材料项目有序推进。资源型城市转型与可持续发展规划、煤矸石综合利用发展自备电规划和焦化、聚氯乙烯两大基地发展规划申报工作进展顺利。科技创新能力逐步增强。内蒙古煤焦化工新材料研究院和氯碱化工技术研究院挂牌成立，全社会R&D经费支出5.4亿元，占GDP比重创历史新高。乌海市被确定为国家级首批循环经济示范城市，连续两次被评为全国科技进步先进市。

　　第三产业快速发展。预计第三产业增加值完成176亿元，增长7.5%。金融服务业持续向好，全市银行各项存款余额达到585.7亿元，增长8.8%;市内外银行各项贷款总额达到598.6亿元，增长22.4%，贷款总额首次高于存款余额。积极营造法制金融生态环境，成立自治区首家金融案件审判合议庭。银行不良贷款率为0.33%，商业信用环境指数居全区第3位。

　　商贸流通业发展步伐加快，格兰云天酒店建成运营，宜化广场、谊君半岛商务中心、红星美凯龙家居等项目基本建成，成功引进万达广场、沃尔玛、月星家居、北京华联等20多家知名商贸企业;宜嘉药品仓储中心开工建设，凯阳建材城主体完工，广东塑料交易所物流交收仓储中心、如意俊安一期项目基本建成。第一粮库“退城进郊”项目投入使用。铁路运输结构调整成效明显，“白货”外运量占比达到56%，较去年提高2个百分点。房地产业运行总体平稳，全年完成投资48亿元，商品房销售面积和销售额分别增长8%和12%。旅游业发展步伐加快，全面推进甘德尔生态文明景区、龙游湾湿地公园等八大旅游景区建设，旅游业总收入完成20.2亿元，增长22%。

　　农业产业化稳步推进。预计第一产业增加值完成5亿元，增长5%。认真落实各项惠农政策，发放　　各类支农惠农补贴资金1.1亿元。蔬菜、葡萄等优势产业进一步发展壮大，蔬菜播种面积完成1.7万亩，新植葡萄4400亩，机场路两侧现代农业示范园和阳光田宇葡萄品种园建设项目进展顺利，花卉市场启动建设。出台了加快葡萄产业发展的意见，葡萄产业品牌竞争力不断增强，汉森葡萄酒荣获第17届柏林国际葡萄酒质量大赛金奖。

　　（三）人居环境得到明显改善。城市规划龙头作用明显。全面推进城市总体规划，城市整体布局更加合理。三区控制性详细规划得到批准，滨河二、三期控制性详细规划及环山湖概念性规划、滨河西区用地规划编制完成，滨河二期中央公园、葡萄主题公园、书法主题广场等规划设计启动实施。城市建设管理力度不断加大。国家重点项目黄河海勃湾水利枢纽工程主体基本完工并实现初期蓄水，护岸及配套工程基本完工。滨河二期房屋征收和土地收储基本完成，基础设施建设全面启动。旧城改造稳步推进，完成房屋征收面积57.2万平方米。城市夜景亮化水平不断提高，全年投入4200多万元完成夜景亮化项目97个。城市配套服务功能进一步完善，供热普及率和燃气普及率分别达到83.2%和83.1%，生活垃圾无害化处理率和生活污水集中处理率分别达到86.1%和93.2%。加强城市精细化管理，数字城管平台正式启动，智能交通信息系统一期完成，乌海市成为全国首批、自治区唯一的智慧城市试点。立体交通网络逐步完善。全年完成投资7.6亿元。海南至乌达重载一级公路建成通车，乌海湖大桥、110国道黄河大桥等项目进展顺利。乌海机场航线网络布局不断完善，“呼乌快线”正式开通，全年旅客吞吐量达到47万人次，增长47.5%。生态建设力度加大。投入生态建设资金23亿元，是历年投入力度最大的一年。海勃湾北部生态涵养区保护管理工作全面启动，重点实施了甘德尔山、城际快速通道等25项绿化工程。全市建成区绿化覆盖率达到40.8%，人均公园绿地面积达到18.4平方米，乌海市被评为全国绿化模范城市。城市水系建设成效显著，甘德尔河二期、机场路两侧8个人工湖全部蓄水，三区城区水系建设进展顺利，乌海市被列为全国首批水生态文明城市建设试点。城市空气质量逐步好转。加快淘汰落后产能，关停白灰立窑企业46户。完成23户重点用能企业能源审计和节能规划，预计单位GDP能耗同比下降3.4%，实施减排项目54个，节能减排完成自治区约束性指标。加大重点行业达标整治和区域联防联控力度，加强环境执法监察，工业企业基本实现达标排放。继续实施露采企业防尘降尘治理工作，矿区粉尘扬尘污染得到一定程度控制。

　　(四)民生保障工作不断加强。全年用于民生社会事业支出占地方公共财政预算支出73.1%。城乡居民收入稳步提高，预计城镇居民人均可支配收入达到27991元，增长10%;农区居民人均纯收入达到13910元，增长12%。政府为民办十二件实事基本落实。就业创业工作成效显著。全年城镇新增就业9232人，完成培训9261人次，发放小额担保贷款突破1亿元，1828人实现成功创业，城镇登记失业率控制在3.6%的较低水平。社会保障体系日益健全。城镇基本医疗保险实现户籍人口全覆盖，基本养老、失业、工伤、生育保险覆盖面稳步扩大。企业退休人员基本养老金标准每月提高152元，达到1712元。最低工资标准提高150元，达到1350元。取暖补贴标准进一步提高。6462名“五七工”和集体企业职工纳入城镇企业职工养老保险范围。工病亡遗属、低保户、低保边缘家庭、优抚对象、计划生育和少数民族困难家庭、残疾人等群体全部纳入城镇居民医保参保范围。社会救助水平不断提高。城镇居民最低生活保障标准提高70元，达到470元;五保对象集中供养和分散供养标准每人每年分别提高1500元和700元，达到8000元和5000元;本科和专科贫困大学生救助标准均提高1000元，达到3500元和2500元;计划生育伤残、死亡家庭扶助标准每人每月分别提高1100元和1365元，达到1200元和1500元;低保残疾人生活困难补贴标准每人每月提高50元，达到100元，各项民生指标均居自治区前列。建立低保对象促进就业工作联动机制，促进有劳动能力的低保对象实现就业。实施惠民殡葬补贴政策，免除低收入困难群体基本殡葬服务费用。深入开展扶贫脱困工作，全年发放困难群众社保补贴、取暖补贴、物价补贴等各类资金4亿元。重视老年人工作，新建改造12个社区综合服务中心，建成10个社区老年人日间照料中心。保障性安居工程稳步推进。新开工建设保障性安居工程13个、5408套，基本建成1.4万套。老石旦、卡布其棚户区搬迁改造任务基本完成，乌达区煤矿棚户区爱民佳苑改造项目获得“中国人居环境范例奖”。

　　（五）社会事业取得全面进步。教育事业全面发展。新建改扩建标准化幼儿园6所，“入园难”问题有效缓解。学校标准化建设和薄弱学校改造工程扎实推进，市一中教学楼、市九中、十六中建成使用。职业教育、民族教育、特殊教育进一步加强，乌海职业技术学院进入自治区首批示范高职院校建设行列。强化师资队伍建设，引进招聘优秀本科生、研究生150名。2013年本科平均录取率达到59.7%，高于自治区15.5个百分点。医疗卫生事业稳步推进。基层医疗卫生机构和公立医院改革持续深化，公共卫生服务体系进一步完善，重大疫情预警监测能力明显增强。医疗卫生基础设施建设不断加强，市人民医院内科楼建成使用，儿童医院改造完成，开工建设精神卫生中心。启动实施基层蒙中医药服务提升工程，建设完成区域卫生信息平台。文体事业繁荣发展。制定出台进一步扶持文体类社会组织加快发展的意见，在全区率先建成启用社会组织孵化基地，入驻文体类社会组织32家。公共文化基础设施进一步完善，青少年创意产业园建成投入使用，书法艺术中心、召烧沟岩画遗址保护工程等项目进展顺利，召烧沟岩画被列为第七批国家级重点文物保护单位。书法城建设深入推进，“翰墨长河”书法美术摄影展在中国美术馆成功举办，“书法五进”工作模式荣获第二批全国公共文化服务体系示范项目创建资格。广播电视 “户户通”和新媒体建设工程全部完成。积极开展体育运动，成功举办首届“乌海湖杯”全国桥牌邀请赛、市第三届全民健身运动会和首届家庭健步走活动。社会管理工作成效显著。全面推行街道社区网格化管理、精细化服务，社区服务水平进一步提高。强化为民便民服务，智慧民生服务平台建设完成，“12345”市民服务热线正式启动。制定出台住宅小区物业服务补贴实施细则，加大物业服务企业扶持力度，物业服务体系逐步完善。社会矛盾纠纷化解多元机制不断健全，人民调解工作稳步推进，群众诉求表达、利益协调、权益保障渠道更加畅通和规范，集中清理和化解了一批信访积案。全面落实企业安全生产主体责任，深入开展隐患排查整治和“打非治违”行动，安全生产形势保持平稳，未发生重特大安全生产事故。深入推进“科技防控城市”建设，加强社会治安综合治理，“平安乌海”建设取得明显成效。人口和计划生育工作保持自治区先进行列。审计工作进一步加强。国防建设、双拥优抚、民族宗教、档案史志、外事侨务、人防、气象、防震减灾、新闻出版、无线电管理和红十字等各项工作取得新成绩。

　　（六）政府自身建设不断加强。民主法制建设稳步推进。自觉接受人大、政协的监督，办理人大代表议案建议、政协委员提案346件，办复率100%。认真听取各民主党派、工商联、无党派人士意见，积极支持工会、共青团和妇联等人民团体开展工作。“六五”普法工作通过自治区中期督导验收。改革创新力度不断加大。实施新一轮行政审批制度改革，行政审批事项由193项减少到87项，行政事业性收费由98项减少到48项，成为全区行政审批事项和行政事业性收费项目最少的盟市。深化政府绩效管理，加强绩效管理考评系统和社会评价系统建设，政府决策更加科学规范。推进城市管理体制创新，城市规划建设监管、土地利用、夜景亮化和户外广告实行集中统一管理;成立滨河新区管理机构，进一步强化滨河新区开发建设管理力度。深化国有资产管理和公共资源交易改革，完成国有资产管理职能划转，实行集中统一管理。推进食品药品监管体制改革，强化监管职能和检测能力建设，食品药品监管水平明显提升。作风转变取得明显成效。认真落实中央、自治区和乌海市关于改进工作作风和密切联系群众的规定，进一步完善市政府工作规则，公务接待更加规范，文件会议数量明显减少。严格控制“三公”经费支出，“三公”经费支出同比下降29.5%。行政办公用房和公务用车清理工作进展顺利。

　　各位代表，过去的一年是乌海发展进程中极不平凡的一年。在经济环境复杂多变、发展面临诸多挑战的形势下，取得这样的成绩实属不易。这是自治区党委、政府和市委正确领导、科学决策的结果，是市人大、政协加强监督、鼎力支持的结果，是全市各族干部群众齐心协力、团结奋斗的结果。在此，我代表市人民政府，向全市广大干部群众和所有关心支持乌海建设发展的各界人士，致以崇高的敬意和衷心的感谢!

　　与此同时，我们也清醒地认识到发展中存在一些问题，主要是：经济总量较小，产业结构单一，工业主导产品初级化、低端化、趋同化特征仍然明显，服务业总体层次不高;带动经济转型升级的重大项目储备不足，固定资产投资后续乏力;城市服务功能还不完善，城市管理水平有待提高，生态建设和环境保护任务艰巨，粉尘污染依然较重，空气质量需进一步改善;物价上涨幅度总体偏高，特别是低收入群体生活压力较大，教育、卫生、文化等优质公共服务资源分布不均;政府自身建设有待进一步加强，部分干部思想观念和工作作风还不适应新形势的要求。对于这些问题，我们将高度重视，采取有力措施，切实加以解决。

　　二、2014年工作安排

　　2014年，政府工作的总体思路是：深入贯彻党的十八大、十八届二中、三中全会，以及自治区党委九届九次、十次全委会议和市委六届七次全委会议精神，全面落实自治区“8337”发展思路和乌海市“一个中心、两个转型、三个率先、五个乌海”发展战略，以“稳中求进、改革创新、转型升级、区域中心、民生优先”为重点，全力推进经济和城市转型升级，加快建设区域中心城市，切实抓好民生改善和社会治理，积极推进生态文明建设，着力做大总量与提高发展质量效益，促进经济持续健康发展、社会和谐稳定。

　　2014年，我们的主要预期目标为：地区生产总值增长10.5%，公共财政预算收入增长9%，全社会固定资产投资增长17%，社会消费品零售总额增长13%，城镇居民人均可支配收入增长11%，农区居民人均纯收入增长13%，节能减排完成自治区约束性控制指标，居民消费价格指数控制在3.5%左右，城镇登记失业率控制在3.6%以内，人口自然增长率控制在7‰以内。实现上述目标，我们要突出抓好以下八个方面工作：

　　（一）突出项目建设，推动经济转型升级。

　　抓好重点项目建设。继续实施重点项目推进制度，加快推进东源科技二期1,4-丁二醇、恒业成二期有机硅、黄河能源三期百万吨焦化等项目建设。扩大煤电一体化电力装机总量，确保东源科技、海欣环保动力车间等项目年内开工，积极争取进入蒙西电网电力外送通道和自治区新型煤化工基地发展规划。深入实施“双百亿”工程，重点扶持德晟、宜化、乌海化工等成为营业收入超百亿元的企业，逐步壮大龙头企业集群。鼓励、引导企业间联合、重组、合作，实现上下游配套、优势互补、市场共赢。加强工业经济监测调度，强化项目跟踪协调，积极帮助企业化解生产经营难题，促进企业产能充分释放。创新招商引资方式，逐步由招商引资转为招商选资，由提供优惠政策转为完善公共服务。加大招商引资力度，重点引进装备制造、新型化工、高新技术、高端服务等产业转型升级项目，力争全年引进到位资金290亿元以上。

　　发展壮大园区经济。加大园区基础设施建设力度，完善水、电、路、蒸汽、污水处理、集中供热、供气等设施，提升园区承载能力。制定出台园区土地管理办法，盘活园区存量土地，促进集约利用。坚持园区产业发展差异化，进一步明确“一区四园”发展定位和产业转型升级的目标路径，制定园区利益分享机制，统筹推进新建大型工业项目和重大技改项目，形成特色鲜明、优势互补的园区经济新格局。积极做好乌海经济开发区申报国家级经济技术开发区和环乌海湖高新技术产业园区工作，争取列入国家低碳产业园区试点，努力创建全国知名品牌示范区。深入落实沿黄沿线经济带发展规划，推动区域合作发展。积极发挥自身产业优势，充分利用周边地区资源，重点发展产业延伸和资源产品深加工项目，促进区域产业融合发展，逐步推动乌海市由煤炭生产中心向资源深加工、研发中心、交易中心转变。

　　提升发展传统产业。围绕煤焦化工和氯碱化工两大基地，积极抓好产业发展规划编制和重大项目建设，推动煤焦、氯碱行业“两化”融合，促进传统产业新型化。煤焦化工重点围绕焦炉煤气综合利用和煤焦油、粗苯深加工，加快产业延伸升级，确保宝化万辰煤焦油深加工、黑猫三兴化工、天津亚东萘系染料等项目年内建成，全力抓好天津渤海精细化工、上海鑫宝精蒽咔唑、北京成城甲醛等项目建设。氯碱化工重点发展PVC、特种PVC、改性PVC，强化烧碱、液氯综合利用，以电石为基础，加快发展PVC、PVA、1,4-丁二醇、丙炔醇等产业链，构建产业多元发展新格局。确保腾龙草甘膦、东药乌海化工丙炔醇、佳瑞米甲基吡啶系列项目投产达效，开工建设海欣一期醋酸乙烯、金斧氰尿酸、台州源宏医药中间体等项目。全面实施创新驱动发展战略，投入工业转型升级、科技创新、非公经济发展等专项资金1.5亿元，引导企业加大科技投入，提高自主创新能力，促进传统产业优化升级。

　　大力发展新兴产业。加快发展清洁能源、精细化工、装备制造、新材料等产业，尽快形成产业竞争新优势，推动新兴产业规模化、支柱产业多元化，重点抓好陕汽新能源汽车二期、镁合金汽车轮毂、海能实业粉煤灰超细环保纤维、双欣活性炭、鼎晶光伏、紫荆贝氏体钢等项目建设。力争全年新兴产业占工业增加值的比重达到30%以上。

　　大力发展非公经济。全面落实国家支持非公经济发展的“新36条”及自治区和乌海市的政策措施，鼓励、支持、引导非公经济发展。逐步实行商事登记制度改革，简化商事主体行政审批，推进工商注册登记便利化。建立中小微企业投融资公司，积极扶持具备条件的民营企业上市融资，切实解决企业融资难题。建立中小微企业创业基地，全力支持民营企业做强做优。

　　（二）突出环湖建设，推进城市转型升级。

　　强化规划龙头地位。将科学化、人性化、制度化和精细化理念贯穿于城市规划建设全过程。树立全市城市规划、土地利用一盘棋思想，实行统一规划、统一审批、统一管理。围绕“一城两中心”城市框架，尽快完善环山湖地区城市用地布局与功能规划、乌海湖周边旅游规划，完成滨河二期城市设计、绿地生态系统、城市出入口、机场净空等规划设计，做好道路、给排水、供热、供气、供电、环卫、绿地等市政公用设施专项规划，逐步建立市域、行业全覆盖的规划体系。强化规划的权威性，严格执行城市规划，保证刚性落实。配合自治区加快实施《乌海及周边地区城镇规划(2010-2030)》，努力把乌海市建设成为呼包鄂城市群和自治区西部的区域中心城市，成为“小三角”区域的核心区和高端区。

　　完善城市服务功能。以滨河二期和环湖区开发为重点，加强城市基础设施建设。完成滨河二期西区7条路网建设，启动滨河二期东区主干道建设，全面配套完善水、电、热、气、通信等管网设施。完成环山环湖绿化、亮化和乌海湖码头、浴场、环湖道路建设，加快推进滨河二期中央公园、环湖景观改造、水利枢纽坝址公园等项目建设，大力引进总部经济、金融、旅游和高端地产项目，把滨河二期打造成功能完善、环境优雅、宜居宜业、休闲娱乐为一体的城市核心区。加快甘德尔山草甸公园、葡萄主题公园、书法主题广场、黄河风情园等项目建设，全面提升城市品位。继续推进旧城区适度开发，加强市政公用基础设施投入，不断完善城市综合服务功能。积极推广绿色建筑，鼓励建设优质成品住宅，努力打造宜居环境。加强交通网络建设，加快建设乌海湖大桥、国道110黄河大桥、巴音赛立交桥，开工建设荣乌高速乌海段、海南至棋盘井城际快速路、公交枢纽站和始末站等工程。

　　提升城市管理水平。全面实施“智慧城市”各项工作，完成智能交通、道路运输指挥平台建设，完善应急管理和应急指挥调度平台，提高信息化管理水平。加强违章建筑监管，做好违章建筑防控拆除工作。加强城市景观亮化建设和户外广告管理，取消和禁用高杆广告和楼体广告，统一规范管理商户牌匾。推行城市人性化管理，合理引导流动摊点规范管理、固定经营。加强便民服务设施建设，全面推动沿街商户和行政事业单位开放内部厕所，继续加大水冲式厕所建设力度。加大城市公交事业投入，制定优惠政策鼓励引导市民乘坐公交出行，购置公交车70辆。加大物业企业扶持力度，进一步提升物业管理服务水平。

　　（三）突出旅游文化产业发展，提升第三产业层次。

　　加快旅游文化产业发展。制定出台文化产业规划和促进旅游业加快发展的意见。加强旅游景区建设，体现文化特色，突出休闲度假功能，加快推进环湖景区、甘德尔生态文明景区等八大旅游景区建设，重点抓好乌海湖景区开发。推进旅游业市场化、规模化、高端化运作，完善提升旅游公共服务体系，精心策划旅游品牌和精品旅游线路，全方位推动旅游产业宣传推介，深化区域合作，努力把乌海市建设成为自治区西部旅游中转地和目的地。以书法文化为龙头，带动文房四宝、书法创作、展览、培训、销售等书法文化产业发展。

　　做大做强金融服务业。支持引进股份制商业银行和期货交易等金融机构。充分发挥政银企对接合作平台作用，认真落实第二届金融超市签约项目。扩大担保公司融资规模，支持村镇银行、民营银行、小额贷款公司、融资性担保公司发展，建立知识产权质押等融资性担保机构风险补偿机制，扩大对中小微企业融资额度。着力将金融服务业发展成为全市支柱产业，加快建设自治区西部区域金融中心。

　　大力发展生产性服务业。培育壮大物流企业，加快发展第三方物流。抓好电子信息产业规划，积极推广电子商务等现代流通方式，加快构建现代流通体系。加快推进广东塑料交易所物流交收仓储中心、源通物流园、同洲物流园二期等物流项目建设，协调推进铁路运输通道的连接和贯通。启动乌海国际物流中心，积极争取设立乌海海关。

　　提升发展商贸服务业。加快推进万达广场、维多利等项目建设，开工建设海北大厦、金鼎国际等大型商业综合体，促进沃尔玛、红星美凯龙家居等商业项目顺利运营，推动商业业态改变，打造面向周边、高水平、差异化的商贸流通中心。

　　（四）突出农业产业化和新农村建设，加快推进城乡一体化。

　　坚持走“生态、高效、特色、精品”现代农业发展之路，大力发展以蔬菜、葡萄为主的特色产业，推动农区现代化和农业产业化。持续抓好“菜篮子”工程，加强蔬菜直销店监管，加快发展设施农业，蔬菜播种面积稳定在1.5万亩左右。积极发展葡萄和葡萄酒庄产业，加快一庄一品、各具特色的葡萄酒庄建设，着力打造机场路两侧葡萄产业观光带，年内新植葡萄1万亩。加快农业产业化进程，支持汉森、云飞、西口风、吉奥尼等农业龙头企业强化品牌建设，继续做大做强。适度发展花卉种植，完成花卉市场建设。抓好万亩滩和盐碱地改造。推动农区土地承包经营权合理流转，发展种养殖规模经营，提高农区居民收入水平。大力推进城乡一体化和新型城镇化建设，有序推进农区居民市民化，逐步实现城乡规划、产业布局、资源配置、基础设施、公共服务、就业社保、生态文明、社会治理等工作的一体化。

　　（五）突出生态文明建设，推进环境质量改善。

　　大力推进生态建设。加强城市水系建设，围绕乌海湖的建成蓄水，建设海勃湾环城水系、东山水系和海南至海勃湾区际水系工程，完成龙游湾湿地公园一期工程，做好三区城市水系和人工湖规划建设，打造水在城中、城在水中、依水而居的宜居城市。抓好绿化带建设，加快推进环乌海防风林带和环乌海湖景观林带建设，进一步加快三区快速通道和京藏高速两侧防风林带建设，加快推进甘德尔山、海勃湾东山、乌达西山和海南恩格尔山绿化工作，打造树在城中、城在林中的生态城市。启动国家园林城市创建，加强城市园林绿化工作，积极实施旧城区绿化提质工程，抓好铁路两侧公园绿地建设。制定采空区、沉陷区、棚户区中长期生态恢复治理规划，实施好骆驼山矿区生态恢复治理。继续推进国家京津风沙源治理、天然林保护等重点林业工程，年内完成绿化面积5万亩。

　　扎实推进节能减排。积极化解产能过剩和淘汰落后产能，充分发挥市场机制作用，实现优胜劣汰。继续强化节能目标责任制考核，深入开展重点用能企业能效对标、能源审计、能源合同管理。全面抓好工业、建筑、交通运输、公共机构等领域节能，大力推广散装水泥和新型墙体材料。抓好主要污染物减排，实施减排项目72个，推进焦化行业提标改造，焦化企业全部实现达标排放。开展清洁生产行动，推进工业余热循环利用和中水回用，积极做好华资、源通、天宇化工等企业申报自治区工业循环经济试点工作。强化煤矸石、粉煤灰等固废综合利用，全力争取《乌海市煤矸石综合利用发展自备电规划》的批复，切实提高工业固废处置利用水平。推进建筑垃圾、餐厨垃圾综合利用和生活垃圾减量分类管理，提高污水处理水平，确保海南区、乌达工业园区污水处理厂完成提标改造，千里山污水处理厂年内投入运行。

　　切实加强环境治理。下大力气抓好工业烟尘、粉尘、扬尘污染治理，对骆驼山沿线露天开采企业实行进矿道路硬化和矿区重点地段覆绿喷淋，降低扬尘粉尘污染。完善企业防尘降尘设施，加大气体回收循环利用。加大环境执法监察力度，坚决清理整治违法排污企业。完善城区和工业园区集中供热、供气管网建设，取缔淘汰燃煤小锅炉。加强区域联防联控，改善大气环境质量。加强海勃湾北部生态涵养区管理，强化饮用水源地保护，加强城市供水检测能力建设，加大城市供水老旧管网改造和净化水基础设施建设力度，确保饮用水质安全。

　　（六）突出民生优先，推进民生和社会事业发展。

　　着力保障和改善民生。按照“高标准、全覆盖、全过程、全方位、可持续”的要求，体现以人为本的理念，力争各项民生事业走在全区前列、达到全国一流城市水平。一要千方百计扩大就业。扎实推进创业就业工程，抓好库区移民农业创业园建设，打造一流的就业创业服务平台。充分发挥中小微企业拉动就业效应，强化职业技能培训力度，不断拓宽就业渠道。加大对就业困难人员的社保补贴力度，统筹解决好高校毕业生、困难家庭、库区移民等群体的就业工作。全年新增城镇就业7500人，失业人员再就业3700人，完成培训9000人次以上，发放小额贷款9000万元以上。二要完善社会保障体系。改革医保支付方式，启动大病保险工作，大病保险补偿比例、医疗保险住院报销比例、门诊报销比例均保持在自治区前列。将“老工伤”人员逐步纳入工伤保险基金统筹管理，严格事业单位参加工伤保险工作。完善社会救助体系，适度提高低保保障水平，逐步提高三无人员、五保对象、孤儿等补助标准。全面开展“市民扶贫脱困工程”，在保障基本公共服务、困难群众基本生活这个底线的基础上，分期分批解决好养老保险、医疗保障、保障性住房等突出问题。完善社会统筹和个人账户相结合的基本养老保险制度，健全多缴多得激励机制。积极推进城乡居民和灵活就业人员社会养老保险工作。加快养老服务体系建设，建成老年养护服务中心;探索社区、居家养老服务模式，在城市规划建设中统筹考虑养老服务功能，规划建设养老示范小区。建立规范安全的住房公积金制度，完善住房公积金提取、使用、监管机制。统筹分类推进棚户区搬迁改造，严格限制拉僧庙、梁家沟、教子沟、苏海图、千里山、三厂等区域开发建设，规划建设政府性平价保障住房，改善不符合棚户区政策的乌海市棚户区居民居住条件;公乌素地区作为控制、提高区，要严格控制开发建设，逐步改善提高区配套设施，有序推进提高区以外区域居民搬迁。全面启动煤矿棚户区二期工程，积极做好独立工矿区棚户区改造前期工作，开工建设各类保障性住房4749套，基本建成9443套，确保符合棚户区政策、具有乌海市户籍的棚户区居民搬得起、住得稳。三要提高居民收入水平。认真落实国家和自治区有关政策，稳步提高企业退休人员基本养老金水平。进一步规范公务员津补贴，全面实施事业单位绩效工资制度。积极推行企业工资集体协商，建立完善职工收入正常增长机制，努力实现职工收入与企业效益同步增长。依法加强市场监管，确保物价总体平稳，切实降低居民生活成本。

　　全面发展社会事业。一要优先发展教育事业。新建、改扩建幼儿园6所，完成“学前教育三年行动计划”幼儿园建设任务，基本解决“入园难”问题。加强校际之间校长和教师交流，强力推进义务教育均衡发展。发展优质特色高中教育，进一步改善办学条件，努力提高普通高中教育质量。加大公共财政支出比例，加强民族教育、特殊教育发展。积极发展职业教育，调整优化专业设置，抓好乌海职业技术学院全区示范高职院校建设项目，开工建设自治区西部(区域中心)职业技能公共实训基地，大力培养实用型人才。继续完善校长聘任制度，加强师德师风建设，强化教育教学管理，提高教育教学质量，努力打造区域教育服务中心。二要大力发展卫生事业。启动国家卫生城市创建。加快全市卫生信息平台建设，不断提高医疗技术和服务水平。完善突发公共卫生事件应急处置机制，提高重大传染病和重大疫情防控能力。做好包括市人民医院在内的健康产业园和市传染病医院新建前期工作，开工建设市蒙中医院二期，建成市精神卫生中心、市职业病防治院，启用市儿童医院和海区中医院，筹建辅助生殖中心和内镜中心。加强卫生医疗交流与合作，着力培养和引进一批专业技术人才。三要加快公共文化和体育事业发展。完成书法艺术中心、召烧沟岩画遗址保护一期工程，逐步实施召烧沟岩画遗址保护二期工程，启动文化产业园项目。支持开展群众自发性文化活动，深入开展广场文化艺术节等惠民文化活动。积极推进“书法五进”全国公共文化服务体系示范项目，完成市民文化活动中心建设规划。完善公共体育设施，加大室内体育场馆建设力度，支持各类体育场馆向社会开放。深入开展全民健身活动，积极参加自治区“十三运”。四要加大社区建设力度。理顺街道、社区和居委会的关系，强化社区基层服务功能。加大社区人才引进力度，逐步提高社区聘用人员待遇。市财政安排1000万元，加强社区基础设施和信息化建设，完善社区公共服务功能，积极申报全国和谐社区建设示范城市。

　　（七）突出改革创新，推进社会治理水平提升。

　　加快推进社会领域改革。进一步理顺政府和社会的关系，激发社会组织活力，逐步形成政社分开、权责明确、依法自治的现代社会组织体制，充分发挥社会组织孵化器作用，重点培育和优先扶持文体类、行业协会商会类、公益慈善类、社区服务类社会组织。加快医药卫生体制改革，推进公立医院改革试点，完善院长聘用制，推行医院全成本核算和绩效管理。继续推进文化团体体制改革，逐步实现政府部门由办文化向管文化转变。创新公共服务供给模式，加快公交、出租车、供热等管理体制改革，大力推进环卫、园林、社区、工勤等领域向社会购买服务。推进公用事业改革，鼓励和引导社会资本参与文化、养老等事业发展。

　　加快推进行政管理体制改革。继续深化行政审批制度改革，全面推行多部门并联办理“一站式”服务模式，打造优质服务环境。进一步完善公共资源交易监管体制和机制，实现公共资源“阳光交易”全覆盖。稳步推进财税体制改革，规范和提高对区级转移支付比例，进一步完善公共财政体系。严格政府举债程序，严把新增债务关口，着力防控债务风险。实行市区机构编制统一规范管理，严格控制财政供养人员和编制人数。加快信息化进程，开工建设全市政务云中心，完善智慧民生服务平台建设，全面建成“12345”便民服务平台，进一步优化政府门户网站功能。推行积分制户籍管理，推动进城农区居民、外来务工人员、流动人口有序实现市民化。加大社会矛盾纠纷排查化解力度，畅通和规范诉求表达、矛盾调处、权益保障渠道，切实加强信访工作，推行网上受理信访制度，及时就地解决群众合理诉求。

　　健全公共安全体系。继续整合食品药品监管职能和检测资源，建立完善权责统一的安全监管体系，保障群众饮食用药安全。深入推进“平安乌海”建设，不断提高平安建设智能化水平，着力打造立体化社会治安防控体系。突出抓好安全生产，加强隐患排查治理和安全预防，坚决遏制重特大安全事故发生。强化应急处置能力，健全防灾减灾救灾机制。完善人口计生利益导向机制，提高家庭发展保障能力。重视残疾人工作。抓好迎接建市40周年和自治区成立70周年大庆项目建设计划，做好“十二五”中期评估，启动“十三五”规划编制工作。进一步做好国防建设、双拥优抚、民族宗教、档案史志、外事侨务、人防、气象、新闻出版、无线电管理和红十字等各项工作。

　　（八）突出职能转变，加强政府自身建设。

　　坚持依法行政。把依法行政作为政府工作的根本要求，提高政府的执政力和公信力。主动接受市委领导，自觉接受人大和政协监督，认真办理人大代表建议意见和政协委员提案，充分听取民主党派、工商联、无党派人士的意见。全面落实《市政府工作规则》各项制度，确保政府工作规范运行。深入推进政务公开，重点推进公共财政预算、公共资源配置、重大项目审批等领域的信息公开，让权力在阳光下运行。

　　坚持求真务实。严格落实中央八项规定、自治区和乌海市配套规定，扎实开展党的群众路线教育实践活动，认真解决存在的“四风”问题。坚持问政于民、问需于民、问计于民，凡涉及群众切身利益的重大决策必须征求群众意见，切实提高政府决策的科学化、民主化水平。狠抓工作落实，明确重点项目和重点工作责任目标，加大督查力度，确保各项工作任务高质量完成。

　　坚持勤政廉洁。厉行勤俭节约，反对铺张浪费，规范公务接待和公车使用，有效压缩行政成本。深入实施政府绩效管理，完善政府考核激励机制。健全财务预算、核准和审计制度，加大重点项目、重点资金和领导干部经济责任审计力度。加强政府采购、招投标管理，严厉打击虚假资质、围标串标、转包挂靠等违法违规行为。严格控制“三公”经费，市区两级部门预算和“三公”经费预算全部公开。严格执行党风廉政建设责任制，切实加大对领导干部权力的监督和制约，有效预防、坚决惩治腐败，营造风清气正、阳光透明的政治环境。

　　各位代表，事业凝聚人心，实干赢得未来。让我们以党的十八届三中全会精神为指引，全面落实自治区“8337”发展思路和乌海市“一个中心、两个转型、三个率先、五个乌海”发展战略，解放思想、坚定信心，大胆创新、扎实工作，为乌海更加美好的明天而努力奋斗!
